# Supplementary material for: Transcriptional Suppression of Diabetic Nephropathy with Novel Gene Silencer Pyrrole-Imidazole Polyamides Preventing USF1 Binding to the TGF-β1 Promoter
Source: Int J Mol Sci. 2021 Apr 29;22(9):4741. doi: 10.3390/ijms22094741 (PMC8125144; doi:10.3390/ijms22094741)
Supplement: Supplementary file 1 [file ijms-22-04741-s001.zip › ijms-1053194-supplementary.pdf]

Supplementary Information

Supplemental Table 1. List of TaqMan Probes

GAPDH		4351317
Osteopontin		Rn00681031_m1
USF1	Probe	[6FAM]AGTAGCCACTGCACCTTCCTGAATCTG[TAM]
	Sense	5′-CAGCCGAAACCGAAGAGGG-3′
	Antisense	5′-GGCGATAGCTACACTAGTTGGG-3′
TGF-β1	Probe	[6FAM]ACAGGGCTTTCGCTTCAGTGCTCACT[TAM]
	Sense	5′-GGCTGAACCAAGGAGACGG-3′
	Antisense	5′-ACGTGGAGTACATTATCTTTGCTG-3′
h-caldesmon	Probe	[6FAM]CCTTTGCTTCCTGCCTCTCACTCCTTTGC[TAM]
	Sense	5′-GGAGGAGGCGAAGGCTAGG-3′
	Antisense	5′-CTCTCTCCGCTCCCTTCTCC-3′

Abbreviations: GAPDH: glyceraldehyde-3-phosphate dehydrogenase, USF1: upstream stimulatory factor 1, TGF-β1: transforming growth factor-β1.

Supplemental Figure 1.

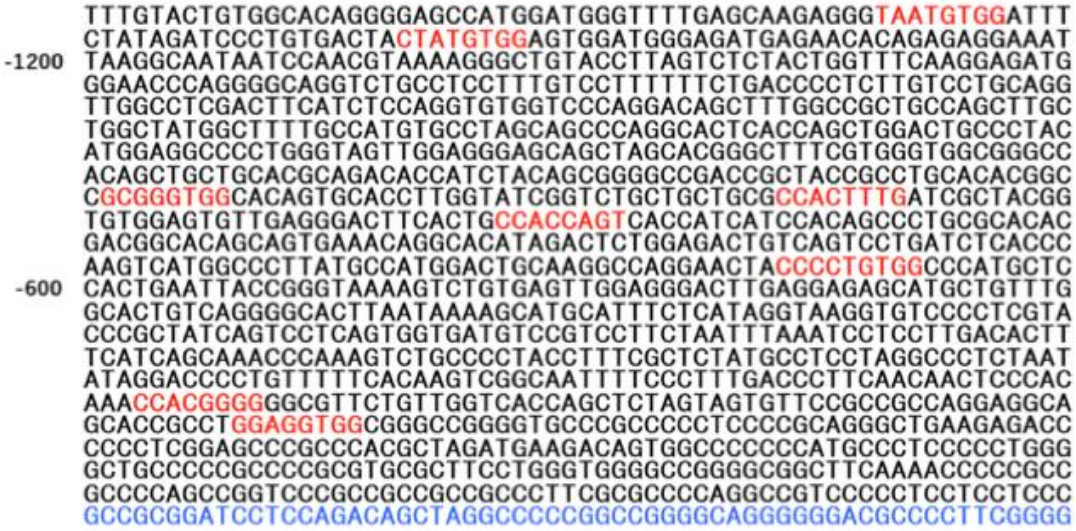

Sequences of rat transforming growth factor-β1 promoter. Red color sequences are USF1-binding E-box regions.

Supplemental Figure 2.

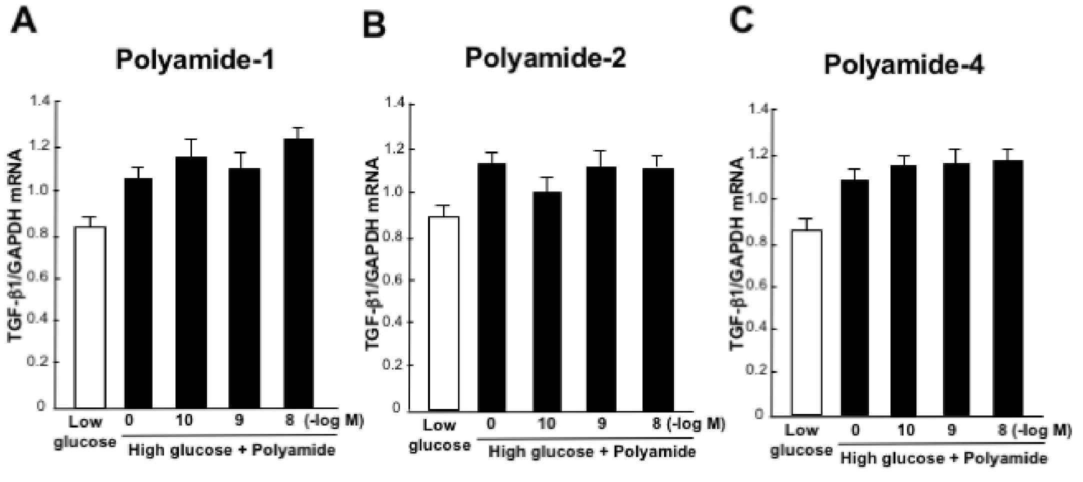

Effects of upstream stimulatory factor 1 pyrrole-imidazole Polyamide-1 (A), -2 (B) and -4 (C) on the expression of transforming growth factor (TGF)-β1 mRNAs in mesangial cells with high glucose stimulation. Data are the mean ± SEM (n = 6).

Supplemental Figure 3.

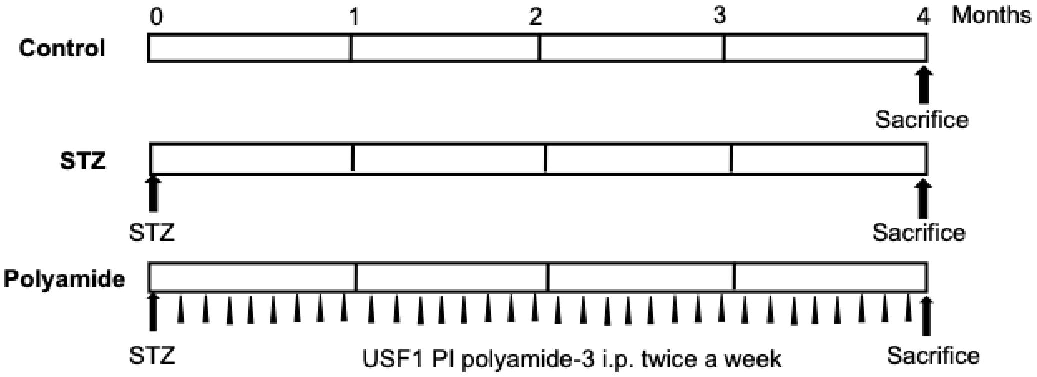

Experimental protocol of the effects of upstream stimulatory factor 1 (USF1) pyrrole-imidazole (PI) polyamide-3 on renal injury in streptozotocin (STZ) diabetic rats. Diabetes was induced in rats by single intraperitoneal injection of STZ. STZ-induced diabetic rats were intraperitoneally injected with 1 mL of 0.1% acetic acid as control rats. Then, 1 mg/kg body weight of USF1 PI polyamide dissolved in 1 mL of 0.1% acetic acid was intraperitonially injected twice a week for 4 months. At 4 months after STZ injection, rats were sacrificed to remove their kidneys.
